# Editorial for the Special Issue: “State-of-Art in Protein Engineering”

**DOI:** 10.3390/biom12070966

**Published:** 2022-07-10

**Authors:** Lada E. Petrovskaya, Dmitry A. Dolgikh

**Affiliations:** 1 Shemyakin & Ovchinnikov Institute of Bioorganic Chemistry, Russian Academy of Sciences, ul. Miklukho-Maklaya, 16/10, 117997 Moscow, Russia; dolgikh@nmr.ru; 2 Department of Biology, M. V. Lomonosov Moscow State University, Leninskie Gory, 1, 119234 Moscow, Russia; 3 Emanuel Institute of Biochemical Physics, Russian Academy of Sciences, Kosygina Str., 4, 119334 Moscow, Russia

This Special Issue of *Biomolecules* demonstrates the almost unlimited possibilities of modern protein engineering in gene expression, protein production and modification, as well as the design and creation of new proteins. The research teams behind the articles included in this Special Issue deal with a variety of proteins of possible medical and/or biotechnological interest, but all of these proteins apply state-of-the-art protein engineering techniques in their research. These techniques include the engineering of chimeric bispecific antibodies, modification of protein stability, enzyme activity and specificity, multiple mutagenesis to analyze the role of amino acid residues in functionally important regions of proteins, etc. 

Developing the theme of the membrane-active properties of cytochrome c, Chertkova R. and coworkers [1] evaluated the ability of amino acid residues from functionally significant regions of cytochrome c to bind to cardiolipin, as well as their membrane-permeable and peroxidase activity. For this purpose, a panel of mutant variants of cytochrome c with multiple substitutions, both in the red Ω-loop and in the universal binding site, was obtained using site-directed mutagenesis. The results of the study of mutant proteins reveal that the conformational lability of the Ω-loop is important for the interaction of cytochrome c with membranes.

Rhodopsin phosphodiesterases (RhoPDEs) possess light-regulated enzyme activities towards cGMP and cAMP and can be used as optogenetic tools. Tian et al. [2] expressed eight RhoPDEs from protists in *Xenopus* oocytes and three of them showed enzymatic activity. RhoPDE from *Choanoeca flexa* exhibited the highest value of light/dark activity ratio. Protein engineering of natural enzymes can be used to improve their properties for optogenetic applications. For example, point mutations in the *Sr*RhoPDE catalytic domain demonstrated the reduced dark activity of this enzyme. 

Rybchenko V. et al. [3] described a new protein engineering approach for IFN-β delivery to ErbB2+ tumors. They developed a crossMab bispecific antibody with the ability to simultaneously bind two proteins, IFN-β and ErbB2+, to create a targeted molecular complex of cytokine and bispecific antibodies. This application of protein engineering opens up the possibility of safely delivering IFN-β in a biologically inactive form during transport, followed by release of the active cytokine in solid tumors.

Extremophilic enzymes are of special interest for biotechnology due to their unusual properties and activity in specific conditions. The Special Issue includes two papers in which new examples of such enzymes are reported, including a novel esterase Est19 from the Antarctic bacterium *Pseudomonas* sp. E2-15 [4] and a cold-active oligo-1,6-glucosidase from a psychrotrophic bacterium *Exiguobacterium sibiricum* (EsOgl) [5]. Est19 demonstrated a high activity in a broad temperature range, from 10 to 60 °C and retained 50% of its activity at 0 °C. EsOgl was active from 20 °C to 40 °C. To increase thermal stability of EsOgl, the authors introduced several proline residues at specific positions of the protein. As a result, a variant with a 12-fold increase in stability at 45 °C was obtained.

In the paper by Khan et al. [6], new biosensors including fluorescent proteins and a transcriptional factor AfArsR are described. The role of specific cysteine residues in the arsenic ion binding was assessed by site-directed mutagenesis. Two types of constructions were obtained using a FRET pair or a single split fluorescent protein, which can be used for monitoring of the toxic arsenic ion in the environment. Additionally, the performance of the obtained biosensors was improved using protein engineering approaches.

In their review, Yurkova M. and Fedorov A. [7] described the role of the bacterial chaperonin GroEL in protein folding, its current and potential use in biotechnology. Chaperones promote proper protein folding and prevent aggregation, so they can be used for protein expression and protein engineering, including co-expression with various target proteins. The authors analyzed data on GroEL from *T. thermophilis* and similar chaperones, their domains and mutants obtained in their laboratory and described in the literature, and discussed their possible biotechnological applications for scientific and biotechnological purposes.

To conclude, this Special Issue of *Biomolecules* includes research and review papers that use the best methods of modern protein engineering to modify and investigate proteins of biomedical importance. The studied proteins include therapeutic antibodies, cytochrome c, GroEL, extremophilic enzymes and others. The data obtained may be of great interest for a better understanding of the structure and structural–functional relationships in proteins and their possible use for the development of new therapeutic and biotechnological forms.
